# Freestyle Local Perforator Flaps for Facial Reconstruction

**DOI:** 10.1155/2015/707065

**Published:** 2015-07-07

**Authors:** Jun Yong Lee, Ji Min Kim, Ho Kwon, Sung-No Jung, Hyung Sup Shim, Sang Wha Kim

**Affiliations:** ^1^Department of Plastic and Reconstructive Surgery, Incheon St. Mary's Hospital, College of Medicine, The Catholic University, 56 Dongsu-ro, Bupyeong-gu, Incheon 403-720, Republic of Korea; ^2^Department of Plastic and Reconstructive Surgery, Uijeongbu St. Mary's Hospital, College of Medicine, The Catholic University, 271 Cheonbo-ro, Uijeongbu 480-717, Republic of Korea

## Abstract

For the successful reconstruction of facial defects, various perforator flaps have been used in single-stage surgery, where tissues are moved to adjacent defect sites. Our group successfully performed perforator flap surgery on 17 patients with small to moderate facial defects that affected the functional and aesthetic features of their faces. Of four complicated cases, three developed venous congestion, which resolved in the subacute postoperative period, and one patient with partial necrosis underwent minor revision. We reviewed the literature on freestyle perforator flaps for facial defect reconstruction and focused on English articles published in the last five years. With the advance of knowledge regarding the vascular anatomy of pedicled perforator flaps in the face, we found that some perforator flaps can improve functional and aesthetic reconstruction for the facial defects. We suggest that freestyle facial perforator flaps can serve as alternative, safe, and versatile treatment modalities for covering small to moderate facial defects.

## 1. Introduction

Facial defects caused by trauma or the surgical excision of tumors can be reconstructed using skin grafts, local flaps, or free flaps. Skin grafting of the face presents limitations because of contractures, poor color, and poor texture match. Local flaps harvested from adjacent tissue are aesthetically ideal for small to moderate defects, but restrictions in mobility and limited availability of the overlying skin and soft tissue are major drawbacks of this technique. Sometimes, resolving the defects necessitates a delayed or secondary procedure [1]. The successful reconstruction of facial defects requires single-stage surgery, wherein tissue that matches the color and texture of the face is harvested [2]. To satisfy these demands, the present study used various perforator flaps for facial defects.

This paper reports our experience in using freestyle perforator flaps for facial reconstruction and discusses the advantages and disadvantages of the aforementioned method.

## 2. Patients and Methods

Between 2004 and 2012, 17 patients underwent facial reconstruction surgery in which perforator flaps were used to cover ear, nasal, perioral, and eyelid defects.

The sample comprised 13 men and four women aged 6 to 73 years old (median age = 63 years).

Retrospective data were obtained from patient demographics, diagnosis, defect location, flap size, source vessel, and complications. These data are shown in Table 1.

To cover the nasal defects, the flaps based on the nasolabial artery or dorsal nasal artery were used. The average flap size was 3.82 cm^2^ (SD = 1.70). Auricular defects were covered by perforator flaps based on the posterior or superior auricular artery. The average flap size was 3.79 cm^2^ (SD = 2.16). The mental perforator flaps for reconstruction of the lower lip defects were based on the mental perforator artery. On average, the flap size was 7.19 cm^2^ (SD = 1.22).

## 3. Surgical Technique and Refinements

All the operations were performed under general anesthesia. Existing skin tumors were excised by tumor resection with wide surgical margins whose appropriateness was confirmed by performing frozen section biopsies during the operations. For skin and soft tissue defects caused by trauma, the wounds were debrided until healthy tissue with pin point bleeding could be seen.

A flap was designed adjacent to a defect based on the amount of tissue that remained for reconstruction. The skin paddle was designed slightly larger than the defect size to enable insetting with minimal tension. A Doppler probe was used to identify the perforators, which were dissected meticulously using loupe magnification. Then, the final perforator was selected by the reliability of the caliber and length among the identified perforators. The flaps were elevated and inset into the defect areas along the axis of the perforator by rotation, transposition, or advancement. If a flap needed rotation for insetting, the perforator artery was dissected more meticulously. If advancement was sufficient, perforator skeletonization was unnecessary. The donor site was closed directly in two layers, the dermis and the skin, with minimal undermining. The flap was sutured in two layers in a tension-free manner, after which a slightly compressive dressing was applied.

## 4. Results

There were seven cases of nasal defects, six cases of auricular defects, three cases of lower lip defects, and one case of eyebrow defect.

In nasal defects coverage, two cases showed venous congestion at the early postoperative stage.

In auricular defects coverage, two patients developed complications: venous congestion and partial flap necrosis.

There were no complications after covering lower lip defects and eyebrow defects with perforator flaps. The donor sites healed completely with direct closure in all cases.

Of four complicated cases, three were reconstructed with rotational flaps and one was treated with advancement flaps.

## 5. Case Presentation

### 5.1. Case 1

**Figure 1:**
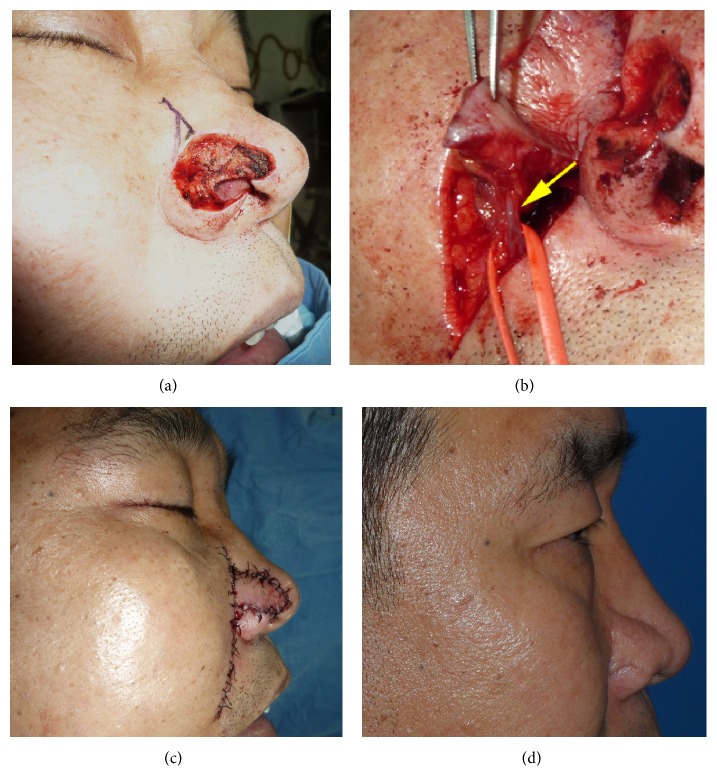
(a) Right ala nasi defect after wide excision of squamous cell carcinoma. (b) The nasolabial artery perforator flap was elevated showing its perforator (arrow). (c) Immediate postoperative photo. (d) Follow-up clinical photo 3 years after surgery.

A 68-year-old male patient with squamous cell carcinoma on his right ala nasi underwent wide excision with a 3–5 mm surgical margin (Figure 1(a)). The defect was reconstructed with a nasolabial artery perforator flap that was slightly larger than the defect. The flap was carefully dissected under loupe magnification and advanced to the defect area (Figures 1(b) and 1(c)). No tumor recurrence was observed during the 3-year follow-up period, and the patient was satisfied with the results aesthetically (Figure 1(d)).

### 5.2. Case 2

**Figure 2:**
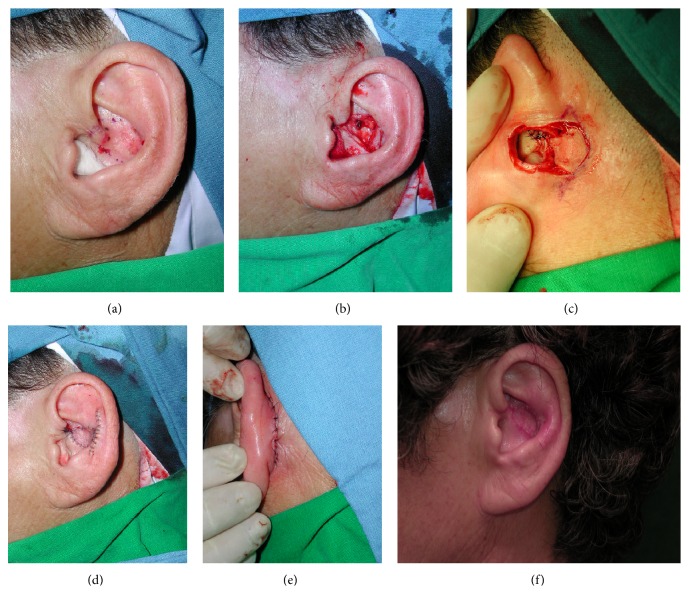
(a) Basal cell carcinoma on the left ear concha. (b) Skin defect after wide excision of tumor. (c) The retroauricular flap and subcutaneous tunnel between the defect and flap are shown. (d) Immediate postoperative clinical photo. (e) The donor site was closed directly. (f) At 5-year follow-up, the patient was satisfied with the level of scarring.

A 65-year-old woman admitted to our department presented with basal cell carcinoma on the left ear concha (Figure 2(a)). The mass was excised with a 3–5 mm surgical margin (Figure 2(b)). To cover the defect, a posterior auricular artery perforator flap was elevated and inset in a flip-flop manner (Figures 2(c) and 2(d)). The donor site was closed directly (Figure 2(e)). The procedure produced aesthetically satisfactory results, and no complications or recurrences were observed during the 5-year follow-up (Figure 2(f)).

## 6. Discussion

Although some reports indicate that freestyle perforator flaps have been used to cover defects of the trunk or extremities, the use of such flaps for facial reconstruction was only recently introduced [3]. With this concept, operations were carried out on the basis of facial artery perforators, nasolabial artery perforators, postauricular artery perforators, or submental artery perforators [2].

The use of facial artery perforator flaps for the reconstruction of perioral defects was first described by Hofer et al. in 2005 [3]. Given the anatomical basis of facial artery perforators, however, the surgery was performed in a freestyle manner. In 2009, the introduction of the perforasome enabled better flap design and clinical usage [4]. Ng et al. identified a reference point where facial artery perforators were consistently found to originate in cadaveric studies [5]. The authors also classified three levels of perforator flaps based on the facial artery subsystem that are used to repair defect of the below the jawline, between the jawline and the nasal alae, and superior to the nasal alae up to the glabella [6].

Cordova et al. introduced the retroauricular island flap based on a postauricular artery perforator for the reconstruction of defects in external ear regions, such as the helix, antihelix, conchal surface, antitragus, and external auditory meatus [7].

A pedicled perforator flap on the face can increase flap mobility and provide a flap that contains only necessary tissue and presents cosmetically satisfactory results.

However, the primary closure of the donor site after flap harvest limits the size and location of the pedicled perforator flap on the face. The best areas for pedicled perforator flap harvest are the neck, nasolabial area, temporoparietal area for island flaps, and occipital area [2]. We reviewed English literature on freestyle perforator flaps for the reconstruction of facial defects (Table 2).

Submental flaps for facial and intraoral defect coverage were first described in 1993. These flaps are based on the submental artery, a branch of the facial artery, and have an anterior neck skin paddle which can be an inconspicuous donor site [8, 9].

Nasolabial flaps were the first true perforator flaps in the face used to reconstruct perioral defects. The flaps are based around the facial artery that provides several perforators between the alar base and mandibular area. It can extend to the lateral cheek and lower eyelid [10, 11].

Superficial temporal artery-based perforator flaps can be used as small hair-bearing flaps for reconstructing the eyebrows and mustache. The superficial temporal artery provides two superficial branches: the frontal and parietal branches. The parietal branches serve as the pedicles of the flap [2].

Furthermore, the superficial temporal artery supplies the auricle and mastoid regions along with the posterior auricular artery. The flap that contains the retroauricular area skin paddle is based on the vascular anastomoses of the superficial temporal artery and posterior auricular artery [12]. The retroauricular flap can be used to cover defects of the nose, ears, eye sockets, eyelid, eyebrow, malar area, and forehead [13–15]. A necessary procedure is to create a subcutaneous tunnel between the pedicle base and the defect area for insetting the flap into the defect.

Occipital artery-based perforator flaps are hair-bearing flaps. The occipital artery originates from the external carotid artery and has three perforator branches: the ascending, transverse, and descending branches [16]. All three perforators can function as reliable vascular pedicles for scalp and neck flaps. The donor site should be covered with a skin graft because of scalp tension. Coverage can extend to the anterior chin, but the flap is more frequently applied to posterior scalp defects.

The perforators are fixed in their locations and supply reliable skin paddles. If a surgeon detects a reliable perforator using a Doppler probe, he/she can use it as a pedicle for flaps that cover other defects on the face. This approach is limited only by the possibility of primary closure of the donor site.

## 7. Conclusion

With the advance of knowledge regarding the vascular anatomy of pedicled perforators in the face, several very useful perforator flaps have been developed. These flaps can improve functional and aesthetic reconstruction for the face.

We suggest that various facial perforator flaps can serve as alternative, safe, and versatile treatment modalities for covering small to moderate facial defects.

## Figures and Tables

**Table 1:** Patient demographics, defect location, flap size, source vessel, and complications.

Number	Age/sex	Location of defect	Source vessel	Size, cm^2^	Complication	Follow-up (month)
1	47/M	Nose	Nasolabial artery	3 × 2	None	12
2	72/F	Nose	Nasolabial artery	2 × 1.5	None	9
3	52/M	Nose	Nasolabial artery	3 × 1.5	None	18
4	62/M	Nose	Nasolabial artery	3 × 2	Congestion	12
5	70/M	Nose	Nasolabial artery	2.5 × 1.5	Congestion	9
6	73/M	Nose	Dorsal nasal artery	2.5 × 1	Partial necrosis	8
7	68/M	Nose	Dorsal nasal artery	1 × 1	None	15
8	70/F	Ear	Posterior auricular artery	2 × 1.5	None	12
9	66/M	Ear	Posterior auricular artery	2 × 1.2	None	12
10	44/M	Ear	Posterior auricular artery	3 × 2.5	Congestion	10
11	63/F	Ear	Posterior auricular artery	1.5 × 1.5	None	36
12	55/F	Ear	Superior auricular artery	1.7 × 2.1	None	10
13	32/M	Ear	Superior auricular artery	2.3 × 1.6	None	18
14	56/M	Lower lip	Mental perforator	4 × 2	None	12
15	70/M	Lower lip	Mental perforator	3 × 2	None	10
16	68/M	Lower lip	Mental perforator	3.5 × 2.5	None	8
17	6/M	Eyebrow	Superficial temporal artery	4 × 1.5	None	48

**Table 2:** Various freestyle perforator flaps used for facial reconstruction.

Originating artery	Flap	Pedicle source	Applications
Facial artery	Submental artery perforator flap	Submental artery	Cheek, perioral, intraoral defect
	Nasolabial flap	Superior and inferior labial artery	Perioral, lateral cheek, lower eyelid
	Lateral nasal artery perforator flap	Lateral nasal artery	Nasal dorsum, ala, side wall
	Angular artery perforator flap	Angular artery	Glabella, inner canthal area
	Buccinator flap	Buccal artery	Intraoral defect

Ophthalmic artery	Supratrochlear artery perforator flap	Supratrochlear artery	Nose, periorbital area
	Supraorbital artery perforator flap	Supraorbital artery	Periorbital area

Superficial temporal artery	Superficial temporal artery perforator flap	Superficial temporal artery	Forehead, periorbital area
	Retroauricular flap	Superficial temporal artery	Ear, nose, eyelid, eyebrow, cheek, forehead
		Posterior auricular artery	

External carotid artery	Occipital artery perforator flap	Occipital artery	Chin
